# Novel device for application of continuous mechanical tensile strain to mammalian cells

**DOI:** 10.1242/bio.023671

**Published:** 2017-03-16

**Authors:** Satoshi Wada, Hiroyuki Kanzaki, Tsuyoshi Narimiya, Yoshiki Nakamura

**Affiliations:** Department of Orthodontics, School of Dental Medicine, Tsurumi University, 2-1-3 Tsurumi, Tsurumi-ku, Yokohama, Kanagawa 230-8501, Japan

**Keywords:** Simple stretch device, Continuous tensile strain, Periodontal ligament cell, Mechanical stress, Osteoblastic differentiation

## Abstract

During orthodontic tooth movement, the periodontal ligament (PDL) is exposed to continuous mechanical strain. However, many researchers have applied cyclic tensile strain, not continuous tensile strain, to PDL cells *in vitro* because there has been no adequate device to apply continuous tensile strain to cultured cells. In this study, we contrived a novel device designed to apply continuous tensile strain to cells in culture. The continuous tensile strain was applied to human immortalized periodontal ligament cell line (HPL cells) and the cytoskeletal structures of HPL cells were examined by immunohistochemistry. The expression of both inflammatory and osteogenic markers was also examined by real-time reverse transcription polymerase chain reaction. The osteogenic protein, Osteopontin (OPN), was also detected by western blot analysis. The actin filaments of HPL cells showed uniform arrangement under continuous tensile strain. The continuous tensile strain increased the expression of inflammatory genes such as *IL-1β*, *IL-6*, *COX-2* and *TNF-α*, and osteogenic genes such as *RUNX2* and *OPN* in HPL cells. It also elevated the expression of OPN protein in HPL cells. These results suggest that our new simple device is useful for exploring the responses to continuous tensile strain applied to the cells.

## Abstract (precis)

**Summary:** Continuous tensile strain from the device changed the cell morphology and increased the expression of inflammatory and osteogenic gene. These effects were similar to those in the PDL during orthodontic tooth movement.

## INTRODUCTION

Cells in the body are usually exposed to several types of mechanical stimulation, such as shear stress (Li et al., 2005), compressive stress (MacKelvie et al., 2003; Tschumperlin et al., 2004) and tensile stress (Thomas et al., 2006). For example, vascular endothelial cells are subjected to shear stress from blood flow (Li et al., 2005), vascular smooth muscle cells are exposed to cyclic stretch resulting from pulsatile pressure (Li et al., 2005), cartilage in human joints is subjected to compressive stress during exercise (MacKelvie et al., 2003), and bronchial epithelial cells undergo tensile and compressive stress by breathing (Thomas et al., 2006; Tschumperlin et al., 2004). Thus, many living cells respond to mechanical strain and adapt to their different physical environments. Several studies have reported that mechanical strain regulates various cellular functions such as proliferation, differentiation, apoptosis and migration in mammalian cells (Banes et al., 1995; Lambert et al., 1998; Petrov and Usherwood, 1994). To investigate the cellular response to mechanical stress, numerous researchers have contrived devices to apply mechanical strain, such as shear stress (Jacobs et al., 1998; Yoo et al., 2014), tensile strain (Beckmann et al., 2014; Kanzaki et al., 2006; Shah et al., 2013; Tsuji et al., 2004; Zhu et al., 2008), compression (Kanzaki et al., 2002; Tschumperlin et al., 2004) and hydrostatic pressure (Swartz et al., 2001), to cultured cells in order to mimic the *in vivo* environment. Much of the mechanical strain in the living body is cyclic in nature, and continuous mechanical strain is only applied in limited situations, such as in orthodontic tooth movement and distraction osteogenesis (Beertsen et al., 1997; Meikle, 2006; Peltomäki, 2009).

In orthodontic tooth movement, continuous mechanical strain is systematically applied to the teeth, which are moved by reconstruction of the periodontal ligament (PDL) interposed between the tooth root and alveolar bone (Beertsen et al., 1997; Meikle, 2006). Cells in the PDL are subjected to continuous mechanical strain and are forced to adapt to the new environment by synthesis and secretion of several cytokines and growth factors (Arai et al., 2010; Baba et al., 2011; Saito et al., 1991; Tsuge et al., 2016). As a result, reconstruction of the PDL and alveolar bone occur both in the tension and compression zones of the PDL (Nakamura et al., 2003; Shimpo et al., 2003; Takahashi et al., 2003, 2006). However, the detailed mechanisms of the cellular response in reconstruction of the PDL and alveolar bone have not been clarified. In particular, the relationship between continuous tensile strain and osteogenic markers in the tension zone of the PDL remains unclear during orthodontic tooth movement.

In order to clarify the molecular regulatory mechanism of tissue reconstruction in the tension zone of PDL during tooth movement, it is necessary to contrive devices in order to investigate the response of PDL cells to continuous tensile strain *in vitro*.

In this study, we contrived a simple device, ‘Cell Extender’ (ver. 3), designed to apply both continuous and cyclic tensile strain to cultured cells *in vitro*. Furthermore, this device offers advantageous cost, size, usability and versatility for experiments with a variety of cell types.

## RESULTS

### Continuous tensile strain from the device apparently influenced the direction of actin filaments in HPL cells

**Fig. 1.**
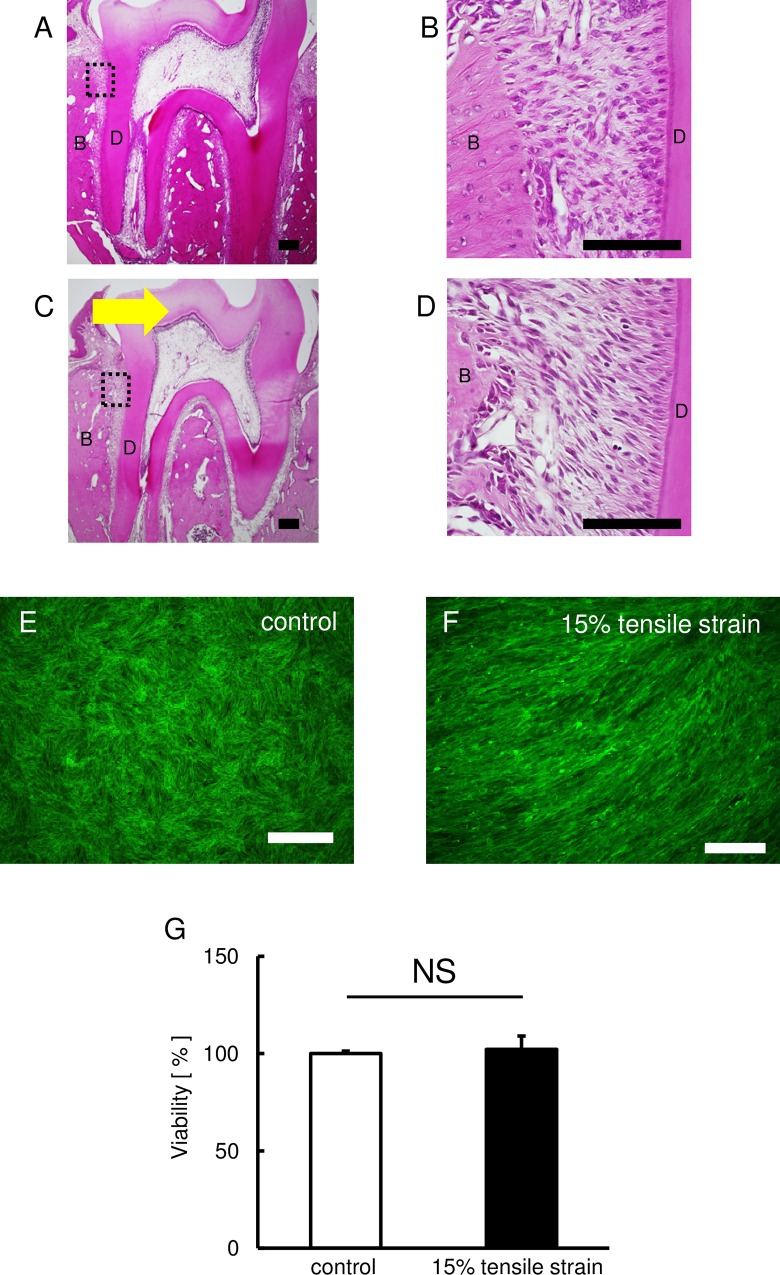
**Effect of continuous tensile strain on the morphology of HPL cells.** (A-D) Images of the PDL stained with H&E stain. Control group (A and B) and 5 days after tooth movement (C and D). B and D are higher magnification images of the boxed area in A and C. Arrow indicates the direction of tooth movement. B: bone, D: dentin. Scale bars: 100 μm. The effect of continuous tensile strain on cell morphology and cytoskeletal was assessed by means of F-actin staining. (E,F) The green fluorescence indicates the F-actin. Representative photographs of the control (E) and stretched HPL cells (F) are shown. Scale bars: 50 μm. (G) The effect of continuous tensile strain from the device on cell viability examined by using cell counting kit-8 (E). Percentage of controls is shown. Mean±s.d.; NS, not significant.

Initially, we observed the tension zone of PDL using perfusion-fixed sections of the PDL of first molar area. The control groups showed that the fibroblasts were scattered in the PDL (Fig. 1A). In contrast, the cellular elements were elongated among the periodontal fibers in the tension zone (Fig. 1B).

Next, we applied continuous tensile strain from the device to HPL cells and examined their cytoskeletal structure, because the cytoskeleton reveals changes in cell morphology. Immunohistochemistry demonstrated that actin filaments were unidirectionally arranged in HPL cells exposed to the continuous tensile strain, though filaments were randomly arranged in HPL cells in the control group (Fig. 1C,D). To examine the effect of continuous tensile strain from the device on cell viability, there were no differences in cell viability between both control and experimental groups (Fig. 1E).

### Continuous tensile strain from the device influenced expression of inflammatory genes in HPL cells

**Fig. 2.**
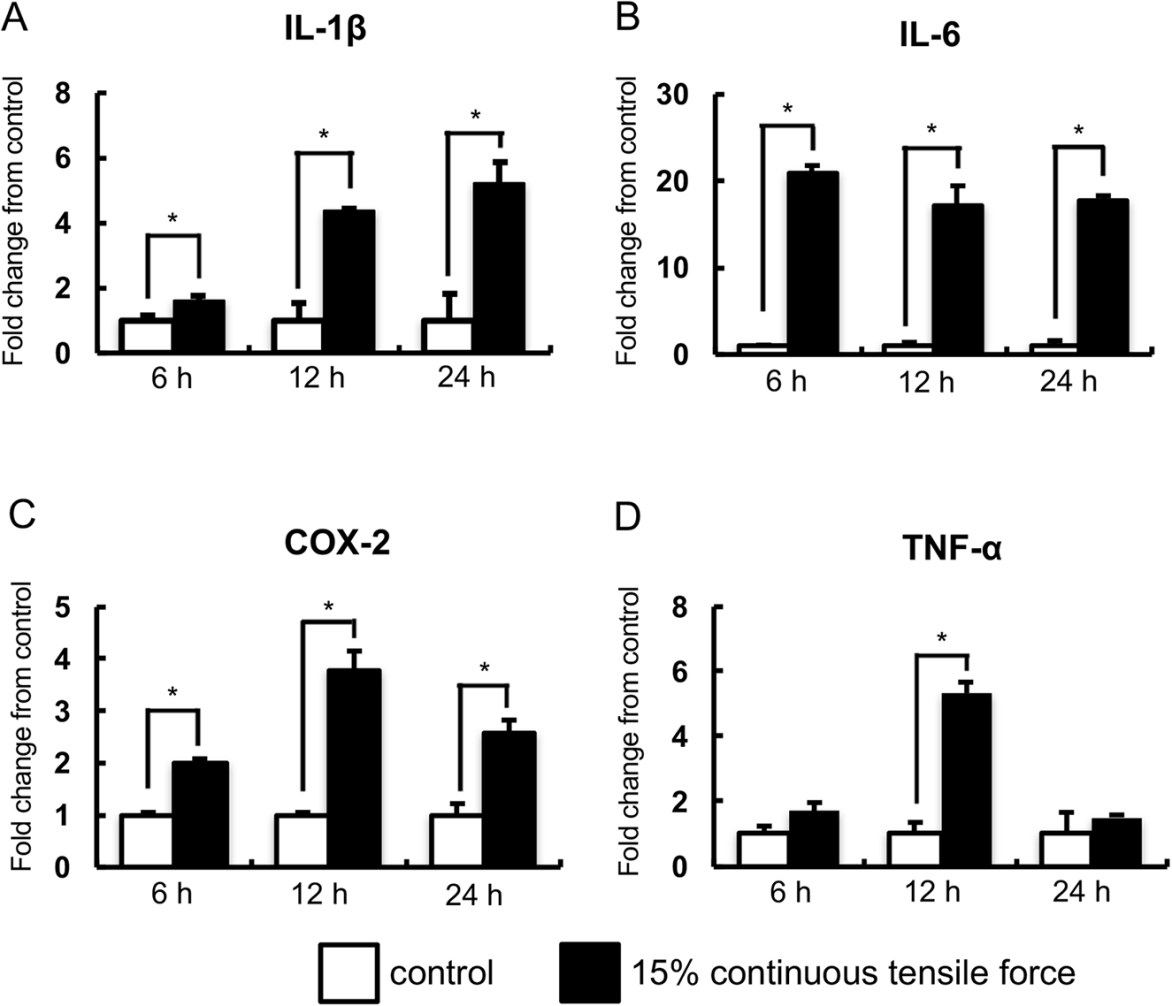
**Real-time RT-PCR analysis of inflammatory gene expression.** The expressions of inflammatory genes in HPL cells with the application of continuous tensile strain at strengths of 15% were examined by real-time RT-PCR. Gene expression was calibrated using the *GAPDH* housekeeping gene, and the values indicate the fold-change from control. The expression of *IL-1β* (A), *IL-6* (B), *COX-2* (C), and *TNF-α* (D) mRNA are shown. Mean±s.d.; *P<0.05 (one-way ANOVA).

It has been reported that the expression levels of inflammatory genes were up-regulated in PDL cells under mechanical strain (Iwasaki et al., 2001; Jacobs et al., 2014; Shimizu et al., 1998; Yamamoto et al., 2006); therefore, we examined the effects of continuous tensile strain on inflammatory gene expression in HPL cells. Real-time RT-PCR analysis revealed that the expression of *IL-1β*, *IL-6* and *COX2* mRNAs was significantly upregulated under tensile strain at 24 h in comparison with non-stretched cultures (Fig. 2A-C). *TNF-α* mRNA was also significantly upregulated in HPL cells at 12 h and its upregulation was reduced to control levels at 24 h (Fig. 2D).

### Continuous tensile strain from the device also influenced osteogenic gene expression in HPL cells

**Fig. 3.**
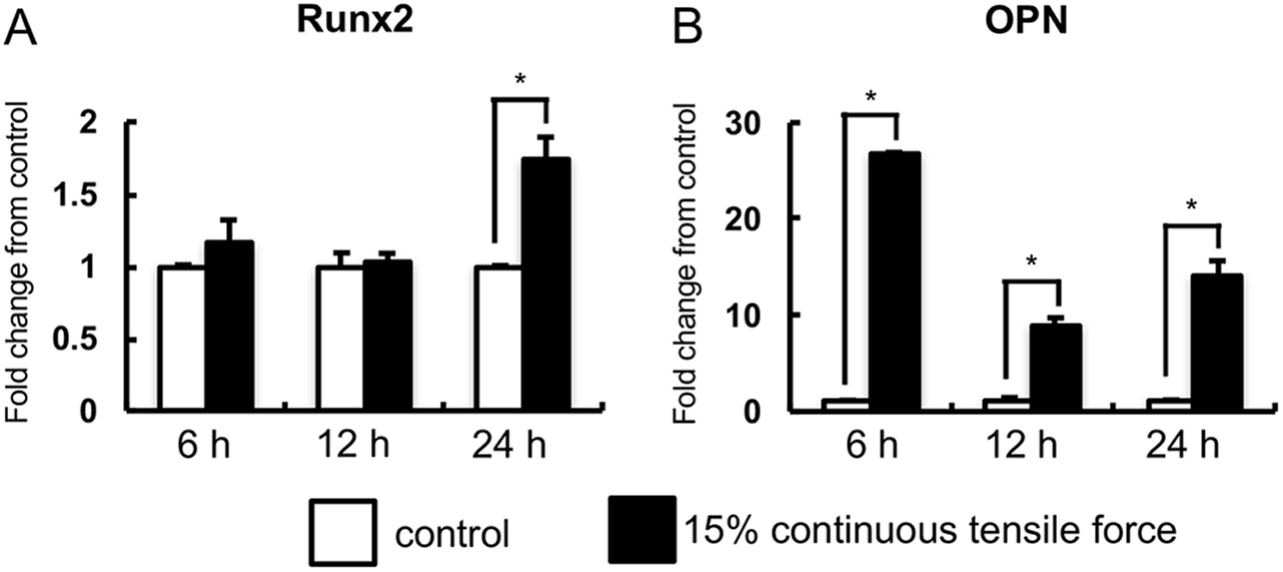
**Real-time RT-PCR analysis of osteogenic gene expression.** The expressions of osteogenic genes in HPL cells with the application of continuous tensile strain at strengths of 15% were examined by real-time RT-PCR. Gene expression was calibrated using the *GAPDH* housekeeping gene, and values indicating the fold-change from control are shown. The expression of *RUNX2* (A) and *OPN* (B) mRNA are shown. Mean±s.d.; *P<0.05 (one-way ANOVA).

Numerous studies have shown that mechanical strain induces osteogenic differentiation of PDL cells by increasing osteogenic genes such as *RUNX2* and *OPN* (Li et al., 2014; Ren et al., 2015; Tang et al., 2014; Zhang et al., 2015). Therefore, we investigated the effects of continuous tensile strain from the device on the osteogenic gene expression in HPL cells. Expression of *RUNX2* was significantly increased by the strain in HPL cells at 24 h when compared to non-stretched HPL cells (Fig. 3A). Expression of *OPN* mRNA was also significantly elevated (Fig. 3B).

### Continuous tensile strain from the device augmented OPN in HPL cells

**Fig. 4.**
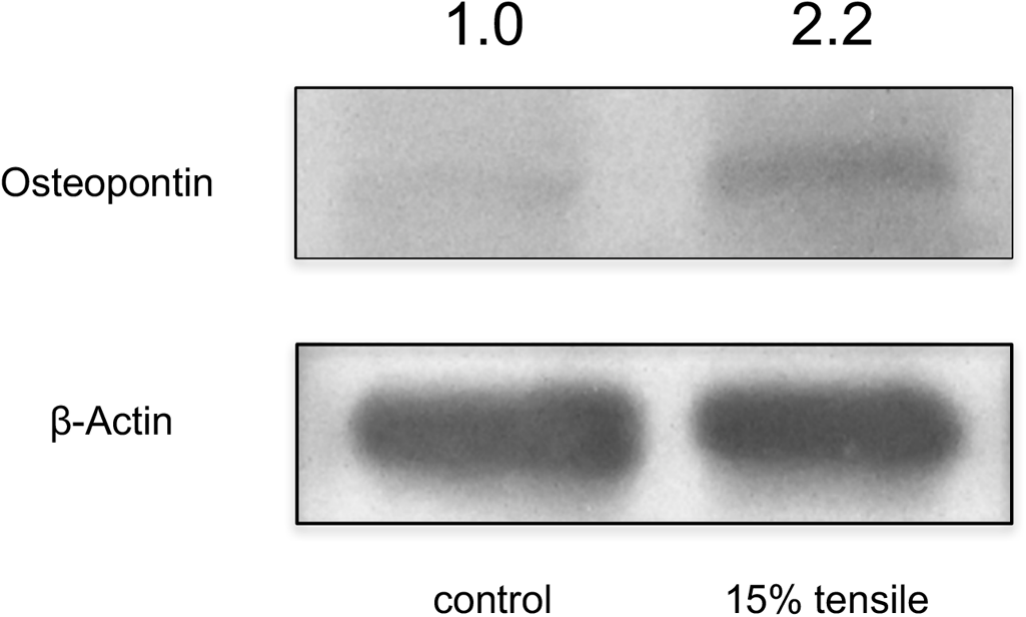
**Western blot analysis for osteopontin expression.** Western blot analysis for osteopontin and β-actin in HPL cells. Representative chemiluminescent image of western blot analysis is shown. The relative band densities in samples from control are shown above each panel.

Next, we examined whether HPL cells induced osteogenic protein under continuous tensile strain by western blot analysis. Western blot analysis for OPN demonstrated that continuous tensile strain augmented OPN in HPL cells (Fig. 4).

### Continuous and cyclic tensile strain from the device induces different responses in HPL cells

**Fig. 5.**
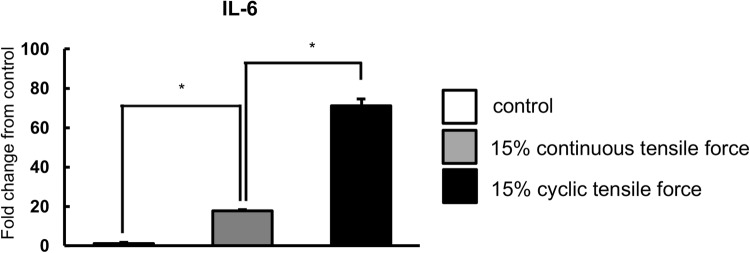
**The continuous and cyclic tensile strain induced different gene expression in HPL cells.** The expressions of inflammatory and osteogenic genes with the application of continuous and cyclic tensile strain at strengths of 15% in HPL cells were examined by real-time RT-PCR. Gene expression was calibrated using the *GAPDH* housekeeping gene, and values indicating the fold-change from control are shown. The expression of *IL-6* mRNA is shown. Mean±s.d.; *P<0.05 (one-way ANOVA).

In order to explore whether there are any differences in cell responses between continuous and cyclic tensile strains, we examined the expression levels of *IL-6* mRNA in HPL cells, as *IL-6* mRNA was markedly increased by continuous tensile strain in HPL cells. Real-time RT-PCR analysis revealed that the expression of *IL-6* was higher in cyclic tensile strain-applied HPL cells than in continuous tensile strain-applied HPL cells (Fig. 5).

## DISCUSSION

In this research, we contrived a simple device, which made it possible to apply both continuous and cyclic mechanical strain to cultured cells. The differences between our device and commercially available devices are size, price, and the mechanism of applying mechanical strain to cells (Banes et al., 1985; Cheng et al., 2015; Hasegawa et al., 2015; Kanzaki et al., 2006; Long et al., 2002; Suzuki et al., 2014; Tsuji et al., 2004). Though commercially available devices are useful for exploring cell responses to cyclic tensile strain, these devices are cumbersome and expensive. Some devices for tensile strain, such as the STREX Cell Stretch System (Strex Inc., Osaka, Japan) (Hasegawa et al., 2015; Suzuki et al., 2014) and the ShellPa mechanical cell stretcher (B-Bridge International, Inc., Santa Clara, CA, USA) (Cheng et al., 2015), elongate the culture well uniaxially (Naruse et al., 1998; Takeda et al., 2006), but the width (orthogonal direction to elongation) of the well becomes narrow, exerting compressive strain on the cells. Our device deforms the flex bottom vertically and consequently imposes only uniaxial tensile strain to cells, in a similar manner as Flexcell Culture Systems (Flexcell International Corp., Hillsborough, NC, USA) (Banes et al., 1985; Kanzaki et al., 2006; Long et al., 2002; Tsuji et al., 2004). Our device and Flexcell Culture Systems are excellent at applying tensile strain to cells. The disadvantage of Flexcell Culture Systems is the cost; they are approximately tenfold more expensive than our device. Taken together, the advantages of our device are compact size, reasonable price, application of uniaxial tensile force without orthogonal compression, and usability.

We demonstrated that PDL cells were elongated along with the periodontal fibers in the tension zone during orthodontic tooth movement. After application of continuous tensile strain from our device, actin filaments in the cultured cell were arranged unidirectionally. Our results are consistent with the response of PDL cells in *in vivo* models, and this indicates that continuous tensile strain from our device reproduces *in vivo* cellular responses in a cell culture system.

In this study, upregulation of osteogenic gene expression was observed in cells under continuous tensile strain (Li et al., 2014; Ren et al., 2015; Shen et al., 2014; Tang et al., 2014; Wei et al., 2008; Zhang et al., 2015). Animal experiments have demonstrated that continuous tensile strain generated by orthodontic appliances also upregulates osteogenic markers in PDL during orthodontic tooth movement (Kawarizadeh et al., 2005; Kim et al., 2012; Watanabe et al., 2008). Therefore, our device induces similar biological effects in the cells.

Continuous tensile strain from the device also upregulated expression of inflammatory genes in the cells. Inflammatory markers were also upregulated in the tension zone of PDL at an early stage of orthodontic tooth movement in the animals (Bletsa et al., 2006; Saito et al., 1991; Tsuge et al., 2016). In this context, our device simultaneously induces upregulation of both osteogenic and inflammatory markers, which is consistent with previous animal experiments.

Although there have been numerous reports of cyclic forces being applied to PDL cells in *in vitro* experiments, very few studies have applied continuous tensile strain to PDL cells (Jacobs et al., 2014). Cyclic tensile strain on PDL cells *in vitro* is used to model the occlusal force during mastication *in vivo* (Li et al., 2013; Pinkerton et al., 2008), which is considerably different from the continuous tensile strain. The difference was clearly demonstrated in the gene expression of inflammatory cytokines, such as IL-6. It has been reported that IL-6 promotes osteoclastogenesis (Udagawa et al., 1995). Expression levels of *IL-6* mRNA are higher after cyclic tensile strain when compared with continuous tensile strain in HPL cells. This finding suggests that the continuous and cyclic tensile strain induces different responses in HPL cells, and that cyclic tensile strain strongly upregulates *IL-6* mRNA expression in HPL cells when compared with continuous tensile strain, thereby promoting osteoclastogenesis. Indeed, other researchers have also reported that the expression levels of inflammatory genes are upregulated by cyclic tensile strain *in vitro* (Jacobs et al., 2014; Mitsuhashi et al., 2011; Saito et al., 1991; Shimizu et al., 1998, 1994; Yamamoto et al., 2006).

In conclusion, we contrived a simple device to apply continuous tensile strain to cultured cells, and the observed biological effects were very similar to those in the PDL during orthodontic tooth movement. Our device would be useful for the investigation of the mechanisms that regulate the response of cells in orthodontic tooth movement.

## MATERIALS AND METHODS

### Animals and experimental orthodontic tooth movement

All experimental protocols were approved by the Institutional Animal Care and Use committee, Tsurumi University (approval numbers; 26A020 and 27A005). All animals were treated ethically, and animal experiments were carried out in accordance with the Guidelines for Animal Experimentation of Tsurumi University, Japan.

Nine-week-old male Wistar rats (total 10 rats; CLEA Japan, Inc., Tokyo, Japan) were used in this study. They were divided into the following two groups. Group 1 consisted of 5 rats, and they wore no orthodontic wire (control group). Group 2 consisted of 5 rats, and they wore orthodontic wire (experimental group). Upper first molars of the experiment groups were moved palatally (0.1 N) with the fixed appliance (Nakamura et al., 2003).

### Histological examination

At the end of experiment, rats were fixed with 4% paraformaldehyde in phosphate-buffered saline (PBS) through their ascending aorta under deep anesthesia with pentobarbital. After perfusion fixation, the orthodontic appliances were removed and the maxillae were dissected and trimmed into smaller blocks containing first molars. The specimens were decalcified with 10% ethylenediaminetetraacetic acid (EDTA) in PBS for 4 weeks, and then washed overnight with 0.1 M PBS at 4°C, dehydrated, and embedded in paraffin. Periodontal tissues from the mesial buccal roots of the upper first molars were examined in serial frontal or cross sections (6 μm-thick), and they were stained with hematoxylin-eosin (H&E).

### Cells

Human immortalized periodontal ligament cell lines (HPL) were a kind gift from Dr Takashi Takata and Dr Masae Kitagawa (University of Hiroshima, Hiroshima, Japan) (Kitagawa et al., 2006).

### Cell culture

HPL cells were cultured in α-modified Eagle's medium (Wako-Pure Chemical, Osaka, Japan) containing 10% fetal bovine serum (Thermo Scientific, South Logan, UT, USA) supplemented with antibiotics (100 U/ml penicillin and 100 μg/ml streptomycin). All cells were cultured at 37°C in a 5% CO_2_ incubator.

### Device design

**Fig. 6.**
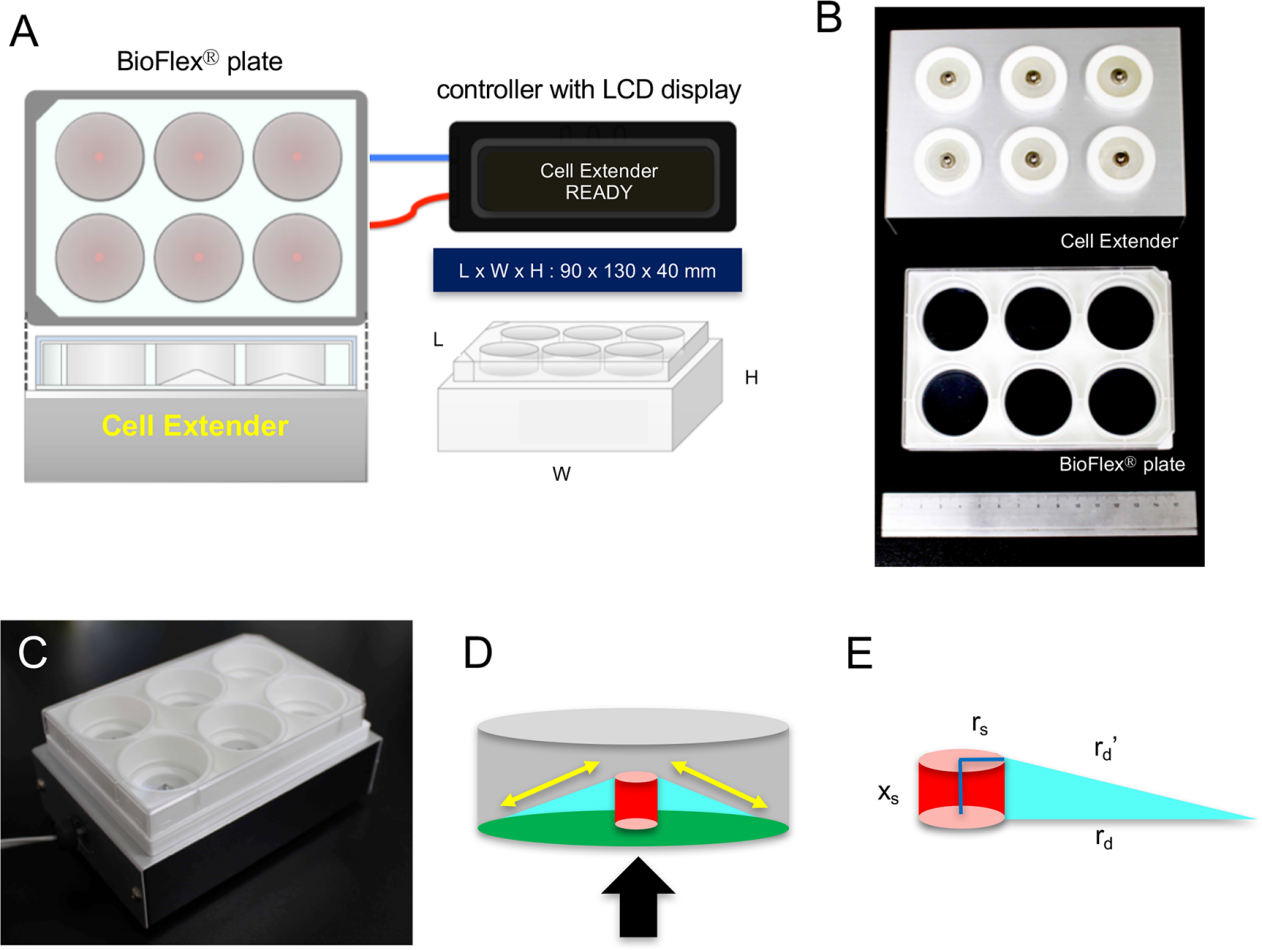
**Details of Cell Extender (ver. 3).** (A) Design of Cell Extender (ver. 3). The device was composed of BioFlex^®^ plate, Cell Extender device, AC adaptor, and controller with LCD display. The size of Cell Extender device is 90×130×40 mm. (B) The Cell Extender device (top) and BioFlex^®^ plate (middle) with the 15-cm scale (bottom). (C) BioFlex^®^ plate positioned on the loading platform of Cell Extender device. (D) Schematic illustration of the flexible membranes at the bottom of BioFlex^®^ plate. When the screw (red) moves upwards, it deforms the flexible membrane of BioFlex^®^ plate (green) and applies strain to the cells on the flexible membrane. Arrow indicates the direction of movement of the screw, and yellow double-arrow indicates the direction of tensile force. (E) Schematic illustration which shows the factors for calculating formula of deformation. *x*_*s*_: screw extension, *r*_*s*_: screw radius, *r*_*d*_+*r*_s_: radius of well bottom, and *r*_*d*_′: change in extension.

The stretched device, Cell Extender (ver. 3, Molcure, Tokyo, Japan), is shown in Fig. 6A. The device is composed of loading platforms and small computer-controlled screws. When the plate with a flexible bottom coated with type I collagen is positioned on the loading platform, the screws are centered beneath the flexible-bottom wells of the plates (Fig. 6B). The screws are controlled by a computer, and deform the plate upward, thereby forcing the flexible bottom of each well to adapt to the screw's surface (Fig. 6C,D). Depending on the height of the screws used, cells are stretched by various magnitudes. The distortion rate of the flexible bottom (*D*) can be expressed as follows:

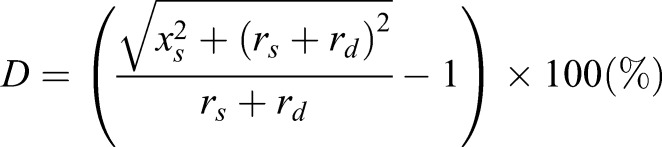
where, *x_s_*: screw extension; *r_s_*: screw radius; *r_d_*+*r_s_*: radius of well bottom; and *r_d_*: change in extension (Fig. 6E). Monitor control enables modification of parameters such as frequency (continuous or cyclic), magnitude (the range from 1% to 30%) and duration (range; 1 s to 24 h) of stretch.

### Tensile strain experiments

HPL cells were seeded at a density of 4.0×10^5^ cells/well on Bioflex^®^ plates (Flexcell^®^ International Corporation, Burlington, NC, USA), which were 35 mm in diameter, 6-well plates with flexible silicone elastomer well bottoms with a total growth surface area of 57.75 cm^2^ (9.62 cm^2^/well). After 24 h, culture medium was replaced and subjected to 15% continuous tensile strain using the device for 6, 12 or 24 h. In some experiments, HPL cells were subjected to cyclic tensile strain (0.5 Hz, 15% elongation). HPL cells were then subjected to microscopic observation or RNA and protein extraction.

### Cell viability assay

The effect of tensile strain using the device was examined by using a cell counting kit-8 (Dojindo, Tokyo, Japan) according to manufacturer recommendations. In brief, HPL cells were plated on 6-well plates and were cultured with 15% continuous tensile strain for 24 h. Then, culture media was exchanged for fresh media containing Cell Counting Kit-8 solution and incubated for 2 h. After incubation, culture supernatant was collected and measured at an absorbance of 450 nm by a plate reader (BioTek Japan, Tokyo, Japan).

### Immunofluorescence studies

After application of tensile strain in HPL cells, cells were fixed with 4% paraformaldehyde in PBS and subsequently permeabilized with PBS including 0.2% Triton X-100 in PBS. After washing with PBS, cells were incubated with ActinGreen™488 ReadyProbes^®^ Reagent (Molecular Probes Inc., Eugene, OR, USA) for 2 h. Stained cells were observed for green fluorescence using a BZ-9000 microscope (Keyence, Osaka, Japan).

### Real-time reverse transcription polymerase chain reaction (RT-PCR) analysis

RNA was extracted using NucleoSpin^®^ RNA (Macherey-Nagel GmbH & Co. KG, Düren, Germany) with on-column genomic DNA digestion in accordance with the manufacturer's instructions. After measurement of RNA concentration, isolated RNA (500 ng each) was reverse transcribed with iScript cDNA-Supermix (Bio-Rad Laboratories, Hercules, CA, USA), and cDNA stock was diluted (10×) with Tris-EDTA buffer. Real-time RT-PCR was performed with SsoFast EvaGreen-Supermix (Bio-Rad Laboratories). PCR primers used in the experiments were as follows: Interleukin-1β (IL-1β) forward, 5′-CACGATGCACCTGTACGATCA-3′ and reverse, 5′-GTTGCTCCATATCCTGTCCCT-3′; Interleulin-6 (IL-6) forward, 5′-AAGCCAGAGCTGTGCAGATGAGTA-3′ and reverse, 5′-TGTCCTGCAGCCACTGGTTC-3′; Cyclooxygenase 2 (COX-2) forward, 5′-TCCTTGAAAGGACTTATGGGTAAT-3′ and reverse, 5′-CTGAATGAAGTAAAGGGACAGC-3′; Tumor necrosis factor (TNF-α) forward, 5′-GACAAGCCTGTAGCCCATGTTGTA-3′ and reverse, 5′-CAGCCTTGGCCCTTGAAGA-3′; Runt-related transcription factor 2 (RUNX2) forward, 5′-AACCCTTAATTTGCACTGGGTCA-3′ and reverse, 5′-CAAATTCCAGCAATGTTTGTGCTAC-3′; Osteopontin (OPN) forward, 5′-ACACATATGATGGCCGAGGTGA-3′ and reverse, 5′-TGTGAGGTGTGTCCTCGTCTGTAG-3′; Glyceraldehyde-3-phosphate dehydrogenase (GAPDH) forward, 5′-GCACCGTCAAGGCTGAGAAC-3′ and reverse, 5′-TGGTGAAGACGCCAGTGGA-3′. Fold-changes in genes of interest were calculated with the ΔΔCt method using GAPDH as a reference gene.

### Western blot analysis

Cells were washed with ice-cold PBS and solubilized in lysis buffer (5 mM EDTA, 10% Glycerol, 1% Triton X-100, 0.1% sodium dodecyl sulfate, and 1% NP-40 in PBS) containing proteinase inhibitor cocktail (Wako). Protein concentrations of protein lysates were measured using the Pierce^®^ BCA protein assay kit (Thermo Fisher), and concentrations were adjusted to be the same. After mixing with 4× sample buffer containing β-mercaptoethanol, samples were heat denatured. Prepared lysates, containing equal amounts of protein, were electrophoresed on a TGX Precast gel (Bio-Rad Laboratories), and proteins were transferred to a polyvinylidene difluoride (PVDF) membrane using a Trans-Blot^®^ Turbo™ blotting system (Bio-Rad Laboratories). After washing with deionized water, membranes were blocked with PVDF Blocking Reagent for Can Get Signal^®^ (Toyobo Co., Ltd., Tokyo, Japan) for 1 h, and were then incubated for 2 h with anti-Osteopontin antibody (Novus Biologicals, Littleton, CO, USA) in Can Get Signal Solution-1 (Toyobo Co., Ltd.). After thorough washing with PBS containing 0.5% Tween-20 (PBS-T), the membrane was incubated for 1 h with horseradish peroxidase-conjugated protein A/G (Thermo Fisher) in Can Get Signal Solution-2 (Toyobo Co., Ltd.), and washed with PBS-T. Chemiluminescence was produced using Luminata Forte (EMD Millipore Corporation, Billerica, MA, USA), and was detected with LumiCube (Liponics, Tokyo, Japan). To confirm the equivalence of loaded protein, the membrane was re-probed with Restore Plus Western Blot Stripping Buffer (Thermo Fisher) for 30 min, washed, blocked, and then blotted in peroxidase conjugated anti-β-actin antibody (Wako).

### Statistical analysis

All data are presented as means and standard deviation from three independent experiments. Differences among independent groups were analyzed by one-way analysis of variance (ANOVA) followed by Bonferroni's multiple comparison using statistical software (ver. 19.0, SPSS STATISTICS^®^, IBM Japan, Tokyo, Japan). *P*<0.05 was considered to be statistically significant.
